# Self-reported limitations in physical function are common 6 months after out-of-hospital cardiac arrest

**DOI:** 10.1016/j.resplu.2022.100275

**Published:** 2022-07-19

**Authors:** Katarina Heimburg, Tobias Cronberg, Åsa B. Tornberg, Susann Ullén, Hans Friberg, Niklas Nielsen, Christian Hassager, Janneke Horn, Jesper Kjærgaard, Michael Kuiper, Christian Rylander, Matt P. Wise, Gisela Lilja

**Affiliations:** a Lund University, Skane University Hospital, Department of Clinical Sciences Lund, Neurology, Lund, Sweden; b Lund University, Department of Health Sciences, Lund, Sweden; c Skane University Hospital, Clinical Studies Sweden – Forum South, Lund, Sweden; d Lund University, Skane University Hospital, Department of Clinical Sciences Lund, Intensive and Perioperative Care, Malmö, Sweden; e Lund University, Helsingborg Hospital, Department of Clinical Sciences Lund Anesthesiology and Intensive Care, Lund, Sweden; f Department of Cardiology, Rigshospitalet, Copenhagen, Denmark; g Department of Intensive Care, Amsterdam UMC, Amsterdam, Netherlands; h Department of Cardiology, Heart Center, Rigshospitalet, Denmark; i Department of Intensive Care, Medical Center Leeuwarden, Leeuwarden, the Netherlands; j Department of Anesthesiology and Intensive Care Medicine, Institute of Clinical Sciences, Sahlgrenska Academy, University of Gothenburg, Gothenburg, Sweden; k Cardiff University School of Medicine, Cardiff, United Kingdom

**Keywords:** Cardiac Arrest, Myocardial Infarction, Patient Reported Outcome Measures, Physical Function

## Abstract

**Title:**

Self-reported limitations in physical function are common 6 months after out-of-hospital cardiac arrest.

**Background:**

Out-of-hospital cardiac arrest (OHCA) survivors generally report good health-related quality of life, but physical aspects of health seem more affected than other domains. Limitations in physical function after surviving OHCA have received little attention.

**Aims:**

To describe physical function 6 months after OHCA and compare it with a group of ST elevation myocardial infarction (STEMI) controls, matched for country, age, sex and time of the cardiac event. A second aim was to explore variables potentially associated with self-reported limitations in physical function in OHCA survivors.

**Methods:**

A cross-sectional sub-study of the Targeted Temperature Management at 33 °C versus 36 °C (TTM) trial with a follow-up 6 months post-event. Physical function was the main outcome assessed with the self-reported Physical Functioning-10 items scale (PF-10). PF-10 is presented as T-scores (0–100), where 50 represents the norm mean. Scores <47 at a group level, or <45 at an individual level indicate limitations in physical function.

**Results:**

287 OHCA survivors and 119 STEMI controls participated. Self-reported physical function by PF-10 was significantly lower for OHCA survivors compared to STEMI controls (mean 46.0, SD 11.2 vs. 48.8, SD 9.0, *p* = 0.025). 38% of OHCA survivors compared to 26% of STEMI controls reported limitations in physical function at an individual level (*p* = 0.022). The most predictive variables for self-reported limitations in physical function in OHCA survivors were older age, female sex, cognitive impairment, and symptoms of anxiety and depression after 6 months.

**Conclusion:**

Self-reported limitations in physical function are more common in OHCA survivors compared to STEMI controls.

**Trial registration:**

ClinicalTrials.gov Identifier: NCT01946932.

## Highlights

• Limitations in physical function are common in cardiac arrest survivors.
• Age and gender are associated with limitations in physical function.
• Cognitive impairment is a predictive variable for physical limitations.
• Anxiety and depression symptoms are associated with physical limitations.
• Physical function should be addressed at follow-up after cardiac arrest.

## Introduction

Out-of-hospital cardiac arrest (OHCA) survivors generally report a good or acceptable health-related quality of life (HRQoL) using questionnaires.1., 2., 3., 4., 5., 6. Compared to other health domains physical aspects seem to be more affected,^2^ but so far limitations in physical function after surviving OHCA have received little attention.7., 8.

Hypoxic-ischemic brain injury increases the risk for long-term physical, cognitive, and emotional problems as well as fatigue in OHCA survivors.1., 6., 8., 9. OHCA survivors admitted to an intensive care unit (ICU) also have an increased risk of post-intensive care syndrome (PICS) including new, or worsening, physical, cognitive and/or mental health problems. Between 25 and 55% of intensive care patients experience one or more of these problems in the long-term. Physical problems may include impaired pulmonary function, muscle weakness and difficulty walking.^10^ Poor physical function after an ICU stay is associated with reduced HRQoL.11., 12.

Physical function is defined as the ability to perform physical movements including simple activities, such as walking, to more complex activities like playing tennis. The most valid and reliable tests of physical function are performance-based.^13^ Physical function can also be measured by self-reports, that are easily administered, and provide valuable information about physical function and experienced physical problems from the patients’ perspective.^11^

Currently there is a lack of information on the extent of limitations in self-reported physical function in OHCA survivors, and how this relates to age, sex2., 14. and comorbidities^15^ well described to be associated with physical function in other populations.^16^ Furthermore, it is unknown how physical function is associated with other factors related to poorer outcome after OHCA as pre-hospital resuscitation variables^1^ and cognitive impairment and symptoms of anxiety and depression.^17^

### Aims

The primary aim of this study was to describe physical function in survivors 6 months after OHCA, and compare it with a group of ST elevation myocardial infarction (STEMI) controls. A second aim was to explore variables potentially associated with self-reported limitations in physical function in OHCA survivors, including sociodemographic characteristics (age, sex, education), pre-event comorbidities (diabetes, hypertension), pre-hospital resuscitation variables (reflected by time to return of spontaneous circulation, ROSC), hospital length of stay (LOS), and the 6 months outcomes of cognitive impairment and symptoms of anxiety and depression.

## Method

### Study design and population

The international multi-centre randomized controlled Targeted Temperature Management at 33 °C versus 36 °C (TTM) trial included 950 adult (≥18 years old) unconscious patients with stable ROSC after OHCA of a presumed cardiac cause, to investigate the effects of target temperature management for mortality and functional outcome.^18^ At 6 months after OHCA, all survivors in the TTM-trial were invited to a structured face-to-face follow-up where information on outcomes were collected.^19^

This cross-sectional study is based on a sub-study involving 20 of 36 original sites in Sweden, Denmark, the Netherlands, the United Kingdom and Italy.^20^ Sub-study participants performed extended assessments at the same time as the main study follow-up. In addition, a control group was recruited at one site in each country and performed an identical follow-up. The control group consisted of patients with a STEMI who received emergency percutaneous coronary intervention and never sustained a cardiac arrest. Controls were matched for country, age (best match), sex and time of the cardiac event (+2 weeks).^20^ The choice of age and sex matched STEMI patients as controls were based on the assumption that they had similar pre-event risk factors and also experienced a traumatic cardiac event, but without the risk of hypoxic-ischemic brain injury and mostly not admitted to an ICU.

The primary intention for the sub-study was to include at least 100 participants in each group (33 °C/36 °C/STEMI controls), recruited with an intended 1:1:1 ratio, in which every second OHCA survivor (33 °C/36 °C) was matched to a control^21^ As there were no differences between the two temperature groups (33 °C/36 °C) in primary and secondary outcome or overall HRQoL19., 21. the OHCA survivors are described as one group in this study, with a 2:1 ratio to STEMI controls. The study designs for both the main TTM-trial and the sub-study, including detailed inclusion and exclusion criteria, are published.20., 22. This study is reported according to the STROBE guidelines^23^ (supplemental material).

### Setting

The participants attended the follow-up at an institution, or in some cases in their own home/nursing home. The examiner was an occupational therapist, a psychologist, a study nurse or a physician that performed the follow-up according to a structured manual^20^ from June 2011 to September 2013.

### Ethical approval, trial registration and informed consent

This investigation conforms with the principle outlined in the Declaration of Helsinki. All participating sites had ethics approval for the TTM-trial with additional approval for this sub-study. Written informed consent was obtained before the 6-month follow-up. The study is registered at ClinicalTrials.gov Identifier: NCT01946932.

### Outcome and outcome measures

#### Main outcome

*Physical Function;* the self-reported health survey SF-36v2® includes the domain Physical Functioning (PF) as one part of physical aspects of health. The SF-36v2® PF domain can be used on its own known as the Physical Functioning-10 items scale (PF-10).^24^ The PF-10 samples three main attributes of perceived limitations in physical function: (1) self-care, (2) mobility and other physical activities, and (3) movements such as lifting and bending.^25^ The 10 items are rated on a hierarchical scale ranging from 1 = not limited to 3 = limited a lot (Table 1). The sum of the 10 items is transformed into a 2009 US general population norm-based T-score using the SF-36v2® Quality Metrics Health Outcome Scoring Software 4.5. A T-score of 50 represents the norm mean. A normal score is ±3 T-scores of the mean at a group level, and ±5 T-scores of the mean at an individual level.^24^ To differentiate between groups a minimal important difference (MID) of 3 T-scores is recommended.^24^ The PF-10 scale has not been previously used as a single instrument in OHCA survivors, but has been used for patients with PICS,11., 26. among the general populations and in patients with various acute and chronic diseases.27., 28. PF-10 exhibits good internal consistency reliability (Cronbach *α* = 0.82) and criterion validity was confirmed amongst older adults.27., 28.

**Table 1:** Self-reported physical function (PF-10) of all OHCA survivors (*n* = 282) and STEMI controls (*n* = 119) 6 months after the cardiac event.

Q: “Does your health now limit you in these activities?”	“If so, how much?”	OHCA	STEMI	*p*-value
		*n* (%)	*n* (%)	
PF1: Vigorous activities	Limited a lot	100 (35.5)	38 (31.9)	
Running, lifting heavy objects, participating in strenuous sports	Limited little	132 (46.8)	61 (51.3)	0.691
	Not limited	50 (17.7)	20 (16.8)	
PF2: Moderate activities	Limited a lot	39 (13.8)	8 (6.7)	
Moving a table, pushing a vacuum cleaner, bowling, or playing golf	Limited little	84 (29.8)	39 (32.8)	0.233
	Not limited	159 (56.4))	72 (60.5)	
PF3: Lifting or carrying groceries	Limited a lot	27 (9.6)	7 (6.0)	
	Limited little	72 (25.5)	34 (29.1)	0.805
	Not limited	183 (64.9)	76 (65.0)	
PF4: Climbing several flights of stairs	Limited a lot	44 (15.6)	21 (17.6)	
	Limited little	98 (34.8)	39 (32.8)	0.851
	Not limited	140 (49.6)	59 (49.6)	
PF5: Climbing one flight of stairs	Limited a lot	20 (7.1)	6 (5.0)	
	Limited little	55 (19.5)	25 (21.0)	0.829
	Not limited	207 (73.4)	88 (73.9)	
PF6: Bending, kneeling, or stooping	Limited a lot	29 (10.3)	13 (11.0)	
	Limited little	97 (34.4)	33 (28.0)	0.393
	Not limited	156 (55.3)	72 (61.0)	
PF7: Walking more than a mile	Limited a lot	58 (20.6)	16 (13.4)	
	Limited little	66 (23.4)	30 (25.2)	0.190
	Not limited	158 (56.0)	73 (61.3)	
PF8: Walking several hundred yards	Limited a lot	25 (8.9)	5 (4.2)	
	Limited little	52 (18.4)	17 (14.3)	0.050
	Not limited	205 (72.7)	97 (81.5)	
PF9: Walking one hundred yards	Limited a lot	15 (5.3)	4 (3.4)	
	Limited little	44 (15.6)	14 (11.8)	0.173
	Not limited	223 (79.1)	101 (84.9)	
PF10: Bathing or dressing yourself	Limited a lot	11 (3.9)	3 (2.5)	
	Limited little	38 (13.5)	9 (7.6)	0.066
	Not limited	233 (82.6)	107 (89.9)	

Abbreviations denote: PF-10 = Physical Functioning-10 items scale, OHCA = out-of-hospital cardiac arrest, STEMI = ST elevation myocardial infarction, PF = physical function.

#### Patient characteristics

Sociodemographic characteristics, pre-event comorbidities, pre-hospital resuscitation variables, and hospital length of stay were collected at the time of hospital admission for the OHCA survivors and at the 6-month follow-up for the STEMI controls.^20^

#### 6-month outcomes OHCA survivors

*Cognitive function*; a dichotomized score of no cognitive impairment (NCI) or cognitive impairment (CI) was based on a combination of three performance-based instruments: the Rivermead Behavioral Memory Test,29., 30. the Frontal Assessment Battery,^31^ and the Symbol Digit Modalities Test.^32^ This dichotomization has been described and used previously (supplement Table A).^17^

*Emotional problems;* the patient-reported Hospital Anxiety and Depression scale (HADS) including two subscales for anxiety and depression symptoms respectively.^33^ Each subscale ranges from 0 to 21, with scores >7 indicating problems.^34^ This cut off was used to create two groups of no symptoms versus symptoms for each subscale separately.

### Statistical methods and analyses

Descriptive statistics are presented with percentages and numbers for binary and categorical variables, for continuous variables as mean and standard deviation (SD) when normally distributed, or median (quartile 1 (Q1) and quartile 3 (Q3)) when non-normally distributed.

Chi-square test was used to detect differences in binary variables and Mann Whitney U-test to detect differences between OHCA survivors and STEMI controls for continuous variables of self‐reported physical function by the PF-10 scale (the sum and the individual items). The transformed norm-based T-scores of the PF-10 were dichotomized into normal (>45) or limited (≤45) physical function for further analyses.

In the binary analyses of potential differences in physical function between OHCA survivors and STEMI controls both unadjusted and multivariable adjusted logistic regression were performed to identify potential influence of pre-event covariates; *age* (years), *sex* (male/female), *education* (<12 years/≥12 years), *hypertension* (no/yes) and *diabetes* (no/yes).

To explore associations between PF-10 and pre-specified variables assumed associated with self-reported limitations in physical function for OHCA survivors, logistic regression was used. First individual univariable logistic regressions were performed for all potential predictors; *age* (years), *sex* (male/female), *education* (<12 years/≥12 years)*, hypertension* (no/yes), *diabetes* (no/yes), *time to ROSC* (minutes), *hospital length of stay* (days), *cognitive impairment* (no/yes), *anxiety symptoms* (no/yes) and *depression symptoms* (no/yes). Then multivariable logistic regression modelling was performed in two steps; the first model included variables presented prior to, or during the OHCA; *age, sex, education, hypertension, diabetes and time to ROSC.* The second model added variables also reflecting the *hospital length of stay* and outcomes 6 months after OHCA; *cognitive impairment, and symptoms of anxiety and depression.*

Results from the logistic regression models are reported as odds ratios (OR) with 95% confidence intervals (CI) and *p*-values. Correlations between the independent variables in the regression models were low to moderate, indicating no problems with multicollinearity.

All tests were two-sided and a *p*-value <0.05 was considered statistically significant. No adjustments for multiple tests were performed as all analyses are considered explorative and hypothesis generating only. Data were computerized and analysed by the IBM Statistical Package for Social Sciences version 26 (Armonk, NY: IBM Corp).

## Results

**Fig. 1:**
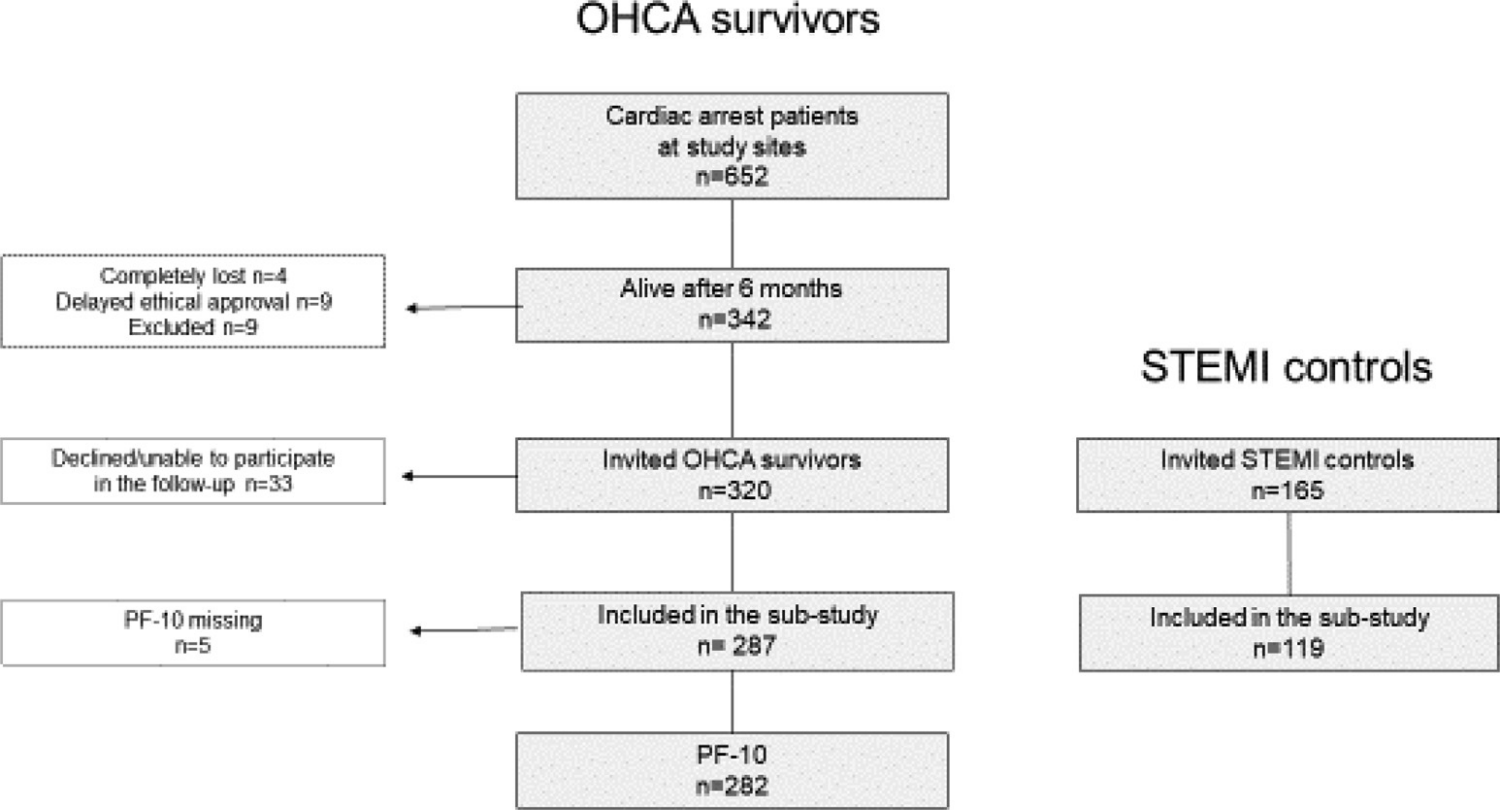
Flowchart for inclusion. Abbreviations denote: PF-10 = Physical Functioning-10 items scale, OHCA = out-of-hospital cardiac arrest, STEMI = ST elevation myocardial infarction.

287 of 320 (90%) OHCA survivors eligible for the extended sub-study participated in the follow-up, together with 119 matched STEMI controls (Fig. 1). The variables for both groups are presented in Table 2. Overall, most variables were similar, although OHCA survivors were slightly younger (62 vs. 64 years) and had a longer hospital stay compared to the STEMI controls (14 vs. 4 days).

**Table 2:** Sociodemographic characteristics, pre-event comorbidities, pre-hospital resuscitation variables (OHCA survivors) and hospital length of stay for all OHCA survivors and STEMI controls, and for OHCA survivors with self-reported normal physical function (PF-10 ≥ 45) and self-reported limitations in physical function (PF-10 < 45).

Variables	OHCA survivors	STEMI controls	OHCA survivors with normal physical function	OHCA survivors with limitations in physical function
	(*n* = 287)	(*n* = 119)	(*n* = 175)	(*n* = 107)
Age years				
Median (Q1, Q3)	62 (54, 69)	64 (57, 71)	60 (51, 66)	64 (58, 73)
Male sex				
*n* (%)	247 (86)	102 (86)	158 (90)	84 (79)
Education				
<12 years *n* (%)	157 (55)	60 (50)	86 (49)	69 (64)
Worked full or part time before cardiac event				
*n* (%)	142 (49)	50 (42)	106 (61)	36 (34)
Pre-event hypertension				
*n* (%)	107 (37)	49 (41)	50 (29)	55 (51)
Pre-event diabetes				
*n* (%)	39 (14)	17 (14)	16 (9)	22 (21)
Location CA at home				
*n* (%)	146 (51)	n/a	85 (49)	61 (57)
Bystander witness				
*n* (%)	266 (94)	n/a	162 (93)	104 (97)
Bystander performed CPR				
*n* (%)	226 (79)	n/a	141 (81)	81 (76)
Bystander defibrillation				
*n* (%)	36 (13)	n/a	24 (14)	12 (11)
CA to ROSC minutes				
Median (Q1, Q3)	20 (14, 30)	n/a	20 (14, 30)	20 (14, 27)
ICU LOS days				
Median (Q1, Q3)	4.5 (3,7)	n/a	4 (3, 6)	5 (4, 8)
Hospital LOS, days				
Median (Q1, Q3)	14 (7, 22)	4 (3, 5)	12 (6, 19)	15 (9, 23)

Abbreviations denote: OHCA = out-of-hospital cardiac arrest, STEMI = ST elevation myocardial infarction, Q = quartile, CA = cardiac arrest, CPR = cardiopulmonary resuscitation, ROSC = return of spontaneous circulation, ICU = intensive care unit, LOS = length of stay.

The PF-10 scale was completed by 282 of 287 OHCA survivors and 119 STEMI controls. At a group level the OHCA survivors indicated limitations in physical function (PF-10 mean 46.0, SD 11.2), but this was not found in the STEMI controls (PF-10 mean 48.8, SD 9.0). There was a statistically significant difference between the two groups (*p* = 0.025), but the mean difference (2.75 T-scores, 95% CI 0.7–4.8) did not reach the threshold of MID (3 T-scores).

In the unadjusted logistic regression analysis, the odds for having self-reported limitations in physical function was significantly higher for OHCA survivors than STEMI controls, OR = 1.74 (95% CI 1.08–2.79, *p* = 0.023), which remained significant in the multivariable covariate adjusted logistic regression analysis (OR 2.01, 95% CI 1.21–3.35, *p* = 0.007).

At an individual level, self-reported limitations in physical function were present in 107 (38%) of the OHCA survivors and 31 (26%) of the STEMI controls (*p* = 0.022). The items of the PF-10 scale where most OHCA survivors and STEMI controls reported limitations were vigorous activities (82% vs. 83%), climbing several flights of stairs (50% vs. 50%) and bending, kneeling or stooping (45% vs. 39%). In addition, almost half of both groups (44% vs. 39%) reported their ability to walk more than a mile (1.6 km) to be limited. There were no statistically significant differences in any of the individual PF-10 items between OHCA survivors and STEMI controls (Table 1).

Descriptive information for OHCA survivors with and without self-reported limitations in physical function are presented in Table 2, Table 3.

**Table 3:** Cognitive impairment, anxiety and depression symptoms at the 6 months follow-up in OHCA survivors with self-reported normal physical function (≥45) and self-reported limitations in physical function (<45).

Variables	Self-reported normal physical function	Self-reported limitations in physical function
	(≥45) (*n* = *175*)	(<45) (*n* = *107*)
Cognitive impairment		
*n* (%)	60 (34.3)	69 (64.5)
Anxiety symptoms		
*n* (%)	27 (5.4)	37 (34.6)
Depression symptoms		
*n* (%)	9 (5.1)	24 (22.4)

Abbreviations denote: OHCA = out-of-hospital cardiac arrest.

There were associations between self-reported limitations in physical function and all pre-defined variables in the univariable analyses, except for time to ROSC (Table 4). Variables with statistically significant differences in PF-10 scores are presented in Table 5. All differences exceeded the value for MID with the greatest difference found for OHCA survivors with and without depression symptoms followed by those with and without cognitive impairment and anxiety symptoms.

**Table 4:** The association between variables assumed important for self-reported limitations in physical function (PF-10 < 45) in OHCA survivors reported by univariable and multivariable logistic regression.

Variables	Univariable model	*p*-value	First multivariable model	*p*-value	Second multivariable model	*p*-value
	OR (95% CI)		OR (95% CI)		OR (95% CI)	
Age						
Years	1.05 (1.03–1.08)	<0.001	1.05 (1.02–1.08)	<0.001	1.07 (1.03–1.10)	<0.001
Female						
Sex	2.55 (1.29–5.03)	0.007	3.88 (1.77–8.55)	0.001	4.35 (1.84–10.32)	0.001
Education						
<12 years	1.91 (1.16–3.14)	0.011	1.44 (0.84–2.48)	0.191	1.32 (0.71–2.43)	0.379
Pre-event hypertension	2.64 (1.60–4.37)	<0.001	1.88 (1.08–3.29)	0.027	1.84 (0.97–3.46)	0.061
Pre-event diabetes	2.57 (1.28–5.16)	0.008	1.81 (0.85–3.85)	0.127	1.72 (0.74–4.01)	0.207
CA to ROSC						
Minutes	0.99 (0.97–1.00)	0.130	0.99 (0.97–1.01)	0.142	0.99 (0.97–1.01)	0.256
Hospital LOS						
Days	1.03 (1.01–1.04)	0.004			1.01 (0.99–1.03)	0.324
Cognitive impairment	3.48 (2.10–5.76)	<0.001			3.14 (1.71–5.77)	<0.001
Anxiety symptoms	3.11 (1.74–5.53)	<0.001			2.33 (1.05–5.17)	0.037
Depression symptoms	5.54 (2.46–12.47)	<0.001			4.31 (1.39 –13.32)	0.011

Abbreviations denote: PF-10 = Physical Functioning-10 items scale, OHCA = out-of-hospital cardiac arrest, OR = odds ratio, CI = confidence interval, CA = cardiac arrest, ROSC = return of spontaneous circulation, LOS = length of stay.

**Table 5:** Comparison of mean PF-10 T-scores between OHCA survivors stratified into groups by different variables as sociodemographic characteristics, pre-event comorbidities, LOS in hospital, cognitive impairment and anxiety and depression symptoms at 6 months follow-up. Minimal important difference (MID) of the PF-10 scores is 3 T-scores.

Variables	Groups	Numbers in each group	PF-10	Mean difference
		(*n* = 282)	Mean (SD)	
Age	≤65 years	181	47.44 (10.45)	
	>65 years	101	43.51 (12.05)	3.93*
Sex	Male	242	46.86 (10.92)	
	Female	40	41.04 (11.63)	5.82*
Education	≥12 years	125	48.11 (9.95)	
	<12 years	155	44.50 (11.66)	3.61*
Pre-event hypertension	No	177	47.69 (10.52)	
	Yes	105	43.24 (11.75)	4.45*
Pre-event diabetes	No	244	46.77 (11.07)	
	Yes	38	41.30 (10.88)	5.47*
Hospital LOS	<14 days	142	47.98 (9.78)	
	≥14 days	140	44.06 (12.17)	3.92*
Cognitive impairment	No	153	49.49 (8.48)	
	Yes	129	41.93 (12.57)	6.31*
Anxiety symptoms	No	209	47.77 (10.17)	
	Yes	64	41.46 (11.76)	6.31*
Depression symptoms	No	243	47.57 (9.95)	
	Yes	33	36.18 (12.94)	11.39*

Abbreviations denote: PF-10 = Physical Functioning-10 items scale, OHCA = out-of-hospital cardiac arrest, SD = standard deviation, LOS = length of stay.

* Indicates a difference between the two means that exceeds what is considered to be a MID.

In the first multivariable logistic regression model (Table 4) including variables prior to and at the OHCA, the significant associations with limitations in physical function remained for age (OR 1.05, *p* < 0.001), sex (OR 3.88, *p* = 0.001) and hypertension (OR 1.88, *p* = 0.027), but not for education or diabetes. In the second multivariable logistic regression model, including also variables reflecting other aspects of 6 months outcome, the effects were consistent for age (OR 1.07, *p* < 0.001) and sex (OR 4.34, *p* = 0.001), with significant associations also for cognitive impairment (OR 3.14, *p* < 0.001), anxiety (OR 2.33, *p* = 0.037) and depressive symptoms (OR 4.31, *p* = 0.011) (Table 4).

## Discussion

This is the first large study reporting detailed information on self-reported physical function in OHCA survivors. The main findings were that self-reported limitations in physical function were common, and that OHCA survivors had significantly more self-reported limitations compared to STEMI controls. Self-reported limitations in physical function were more common in OHCA survivors who were older, female, and had cognitive impairment, and symptoms of anxiety and depression.

A previous study reported that cardiac arrest survivors, had similar limitations in physical function as other general ICU survivors. Both groups had significantly lower scores compared to matched individuals from the general population.^35^ It is likely that the ICU stay itself may be important for the OHCA survivors' physical function. In this study, we included OHCA survivors who were all unconscious and admitted to an ICU. We compared the results to STEMI controls, who were admitted to the hospital, but had not been unconscious at an ICU. The STEMI controls had shorter hospital length of stay compared to the OHCA survivors. Although the OHCA survivors had a statistically significant more limitations in physical function the differences at the individual items of the PF-10 scale were generally small. Few OHCA survivors and STEMI controls were limited in self-care (4% vs. 3%) or household activities (10% vs. 6%), while over 80% in both groups had problems performing vigorous activities.

That older age increased the risk for limitations in physical function was expected and has been described previously in both OHCA survivors^2^ and in the general population.^16^ That sex had an influence on self-reported physical function is also in agreement with a previous study of the general population, where women reported poorer overall PF-10 scores.^16^ Importantly, when comparing our results to sex-matched norm-data for the PF-10 scale,^24^ both the OHCA men and OHCA women reported more limitations in physical function than expected, and the differences exceed the threshold for MID. This result is in line with a French study that found a significant difference in physical function between OHCA survivors and the general population, when matched for age and gender.^36^ This indicates that limitations in physical function in OHCA survivors cannot be explained by age and sex only.

That OHCA survivors with a higher level of education more often had self-reported normal physical function compared to OHCA survivors with lower levels of education is interesting. Persons with a high level of education generally pursue professions that are less physically demanding,^37^ which may lead to less experienced problems. The interactions between education level, working status and self-reported physical function in OHCA survivors are likely complex and needs to be further investigated.

OHCA survivors that reported limitations in physical function more often had pre-event hypertension compared to those without limitations (51% vs. 29%). Interestingly, at a group-level OHCA survivors without hypertension reported normal physical function. Hypertension is a strong, modifiable risk factor for the development of cardiovascular diseases, and physical activity is a non-pharmacological approach to prevent hypertension.^38^ It is likely that self-reported limitations in physical function may affect the ability to perform physical activities and indirectly the blood pressure, but this association needs to be further explored.

We found no significant differences in pre-hospital resuscitation variables between the groups with and without self-reported limitations in physical function. This is in line with a previous study where pre-hospital resuscitation variables were not correlated to SF-36 scores including physical function.^36^

Two thirds of OHCA survivors with self-reported limitations in physical function had cognitive impairment compared to one third of the OHCA survivors without self-reported limitations. Boys et al showed that OHCA survivors with cognitive impairments had lower exercise capacity.^39^ This indicates that these two problems may often coexist.

Symptoms of anxiety and depression were significantly higher in OHCA survivors experiencing limitations in physical function. This is in line with a previous study that concluded that lower levels of physical function was likely to lead to symptoms of depression and anxiety.^40^ Anxiety and depression may also decrease the motivation for physical activity, thereby affecting physical function. In a study of cardiac rehabilitation participants, less symptoms of anxiety and depression were associated with increased physical activity.^41^ After a cardiac event, physical activity is an important feature of both cardiac rehabilitation and secondary prevention to promote both cardiovascular and mental health.^42^

A validation study from the United Kingdom found self-reported limitations in physical function by the PF-10 scale to be related to poorer physical performance in an elderly population.^16^ However the PF-10 is not validated for OHCA survivors or younger adults. We found no problems with floor or ceiling effects for the PF-10, few OHCA survivors (3%) obtained the lowest score and none the highest, and with a wide range in the reported scores (14.9–57.0).

A limitation is that we only used a self-reported questionnaire of physical function in this study. The correlation between self-reported and objective measures of physical function in OHCA survivors is unclear.^43^ The results from self-reports however allow for identification of patients with potential limitations in physical function, who could benefit from physical therapy or other exercise interventions.^13^

Another limitation is that we do not know if any control was admitted to ICU, and we can therefore not exclude PICS-related consequences. However, since PICS is typically associated with prolonged ICU stay,^44^ and the hospital LOS was much shorter for the STEMI controls compared to the OHCA survivors, it is unlikely that this would have major effects on the results. For forthcoming studies, it would be interesting to investigate physical function after OHCA in relation to an ICU control group known to have PICS related problems.10., 11., 12.

The updated European guidelines post resuscitation care recommend greater emphasis on functional assessments of physical impairment before discharge, but in the recommended follow-up after discharge there is less attention to physical limitations.^7^ Our results highlights the importance of identifying those OHCA survivors with limitations in physical function, who may benefit from rehabilitation efforts.

## Conclusions

Self-reported limitations in physical function were more common in OHCA survivors compared to STEMI controls. The most predictive variables for self-reported limitations in physical function in OHCA survivors were older age, female sex, cognitive impairment, symptoms of anxiety and depression after 6 months.

## Funding

This work is supported by grants from The Skane University Hospital Foundations 2019-o000032, 2019; The Skane University Hospital Vårdakademin 2018; Hans-Gabriel and Alice Trolle-Wachtmeister Foundation for Medical Research 2019 and the Gyllenstierna-Krapperup Foundation 2016-0051, 2016. The study sponsors have no involvement in the study design, in the collection of data, or in the forthcoming analyses and interpretation of data, writings of manuscript of manuscripts or in the decisions to submit manuscripts for publication.

## Declaration of Competing Interest

The authors declare that they have no known competing financial interests or personal relationships that could have appeared to influence the work reported in this paper.

## Data Availability

The data that support the findings of this study are available from the corresponding author on reasonable request.
