# Supplementary material for: Self-reported limitations in physical function are common 6 months after out-of-hospital cardiac arrest
Source: Resusc Plus. 2022 Jul 19;11:100275. doi: 10.1016/j.resplu.2022.100275 (PMC9508620; doi:10.1016/j.resplu.2022.100275)
Supplement: Supplementary data 1 [file mmc1.docx]

**Supplement**

**Table A:** Categorization of cognitive impairment.

|  | **Description** | **Abbreviation** | **RBMT** | **FAB** | **SDMT** |
| --- | --- | --- | --- | --- | --- |
| **No cognitive impairment** | Normal score on all three assessments or low/mild disabilities at one or more tests | OHCA-NCI | <16 | >12 | >-1.5 |
| **Cognitive impairment** | Moderate/severe disabilities at one or more tests | OHCA-CI | ≤16 | ≤12 | ≤-1.5 |

Description of categorization into two groups of cognitive outcome based on the cognitive assessments RBMT, FAB and SDMT. For this study, OHCA survivors with cognitive impairment (CI) were separated from those with no cognitive impairment (NCI). Abbreviations denote: OHCA-NCI= out-of-hospital cardiac arrest survivors with no/mild cognitive impairment, OHCA-CI= OHCA survivors with moderate/severe cognitive impairment. RBMT= Rivermead Behavioural Memory Test, FAB= Frontal Assessment Battery, SDMT= Symbol Digit Modalities Test.

**Table B:** Sociodemographic characteristics, pre-event hypertension, pre-event diabetes and hospital LOS for all STEMI controls, and for STEMI controls with self-reported normal physical function (PF-10 ≥45) and with self-reported limitations in physical function (PF-10 <45).

| **Variables** | **STEMI**  **controls**  (n=119) | **STEMI controls with normal physical function** (n=88) | **STEMI controls with limitations in physical function** (n=31) |
| --- | --- | --- | --- |
| **Age** years  median (Q1, Q3) | 64 (57, 71) | 64 ( 57, 70) | 66 (55, 72) |
| **Male gender**  n (%) | 102 (86) | 77 (88) | 25 (81) |
| **Education**  <12 years n (%) | 59 (50) | 40 (45) | 20 (65) |
| **Worked full or part time** before cardiac event n (%) | 50 (42) | 38 (43) | 12 (39) |
| **Pre-event hypertension**  n (%) | 49 (41) | 35 (40) | 14 (45) |
| **Pre-event diabetes**  n (%) | 17 (14) | 9 (10) | 8 (26) |
| **Hospital LOS,** days  median (Q1, Q3) | 4 (3, 5) | 4 (3, 5) | 4 (3, 7) |

Abbreviations denote: STEMI = ST elevation myocardial infarction, Q = quartile, LOS = length of stay.
